# Supplementary material for: Identification of photoperiod-induced specific miRNAs in the adrenal glands of Sunite sheep (Ovis aries)
Source: Front Vet Sci. 2022 Jul 22;9:888207. doi: 10.3389/fvets.2022.888207 (PMC9354845; doi:10.3389/fvets.2022.888207)
Supplement: Supplementary file 1 [file Data_Sheet_1.ZIP › Supplementary file/miRDeep2 file.zip!/miRDeep2 file/Know_Structure/LP42A3/oar-mir-412.pdf]

miRBase precursor : oar-mir-412
Total read count : 969
oar-miR-412-3p read count : 951
oar-miR-412-5p read count : 10
remaining reads : 8

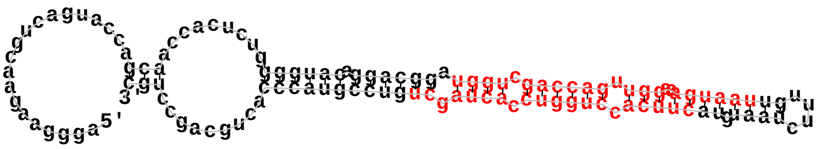

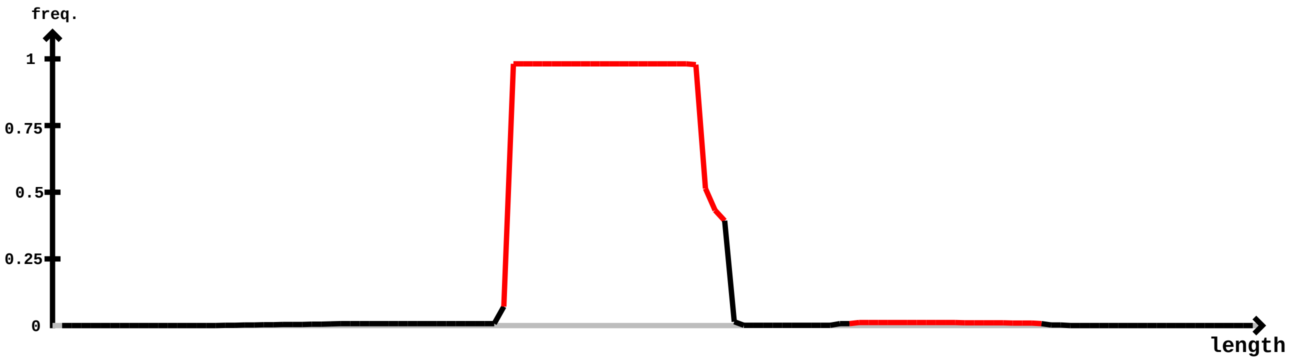


```
                                                                                                     oar-miR-412-5p
                                              oar-miR-412-3p
5'- agggaagaacgucaguaccagcaaccacucugggguacaggacggauggucgaccaguuggaaaguaauuguuucuaauguacuucaccugguccacuagcuguccguacccacugcagccugc  -3'   exp
    ..........(.((((..(((........)))((((((.((((((.((((.((((((.(((..((((((((....)))).))))))).))))))).)))).)))))))))))))))))).......   reads     mm
    ................accagcaaccacucugggguacaggacgga..............................................................................   1         0
    ..................cagcaaccacucugggguacaggacgg...............................................................................   1         0
    ....................gcaaccacucugggguacaggacgg...............................................................................   1         0
    ......................aaccacucugggguacaggacgga..............................................................................   1         0
    .........................cacucugggguacaggacgga..............................................................................   1         0
    ...........................cucugggguacaggacgga..............................................................................   1         0
    ............................ucugggguacaggacgg...............................................................................   1         0
    .............................................auggucgaccaguuggaaag...........................................................   1         0
    .............................................auggucgaccaguuggaaagu..........................................................   52        0
    .............................................auggucgaccaguuggaaagua.........................................................   9         0
    .............................................auggucgaccaguuggaaaguaa........................................................   1         0
    .............................................auggucgaccaguuggaaaguaau.......................................................   1         0
    .............................................auggucgaccaguuggaaaguaauu......................................................   1         0
    ..............................................uggucgaccaguuggaaag...........................................................   2         0
    ..............................................uggucgaccaguuggaaagu..........................................................   398       0
    ..............................................uggucgaccaguuggaaagua.........................................................   70        0
    ..............................................uggucgaccaguuggaaaguaa........................................................   37        0
    ..............................................uggucgaccaguuggaaaguaau.......................................................   367       0
    ..............................................uggucgaccaguuggaaaguaauu......................................................   12        0
    .....................................................................uguuucuaauguacuucaccuggu...............................   1         0
    ................................................................................uacuucaccugguccacu..........................   1         0
    ................................................................................uacuucaccugguccacuagc.......................   2         0
    ................................................................................uacuucaccugguccacuagcu......................   3         0
    ..................................................................................cuucaccugguccacuagcu......................   2         0
    ..................................................................................cuucaccugguccacuagcugu....................   2         0
```
